# Notes from Past Meetings of the Lippe Society

**Published:** 1890-01

**Authors:** 


### NOTES FROM PAST MEETINGS OF THE LIPPE SOCIETY.

The late Dr. Ad. Fellger, of this city, had a vast experience in the treatment of syphilis, and was eminently successful in cases in which much Mercury had been given. Having been a surgeon in the German army for several years he was able to appreciate the difference between old-school empiricism and the law of the similars.

At a meeting of the Lippe Society, in April, 1881, Dr. Fellger said, in commenting on a paper on syphilis which had been read, that "the secondary symptoms of that affection might appear in two months, or it might be forty years." He then related a case of a man in the German army, who at sixty years of age had bubo in the groin which destroyed all the surrounding structures down to the intestines. Another case occurred in Philadelphia, in which the same condition was present. The odor was horrible, and the patient's wife refused to attend him. In this case the symptoms appeared forty years after the primary symptoms.

"You will sometimes find in the neighborhood of a Hunterian chancre a hard lump, which sooner or later spreads, but which has no direct connection with the chancre. The chancre will not heal if this node be present. Dr. Fellger had seen such cases horribly treated with immense doses of Mercury, but without any favorable result. The only good that came of them was the knowledge of the effects of Mercury that he had gained. There was stomatitis, ulcers in the various parts of the throat, swollen, ulcerated tongue, spasmodic cramps, inflammation of the skin, with burning, much perspiration, and great restlessness at night.

"The remedy for painful chancres is Merc-jod.; for painless chancres, Iodine. Where, in the latter stages the hair falls from the head, and grows more luxuriantly on other parts, Lycop. is generally the remedy. Falling out of eyebrows calls for Selenium. The stool of Mercury is diarrhœic, there is much straining and a little blood is passed; the blood occurs in drops. In the diarrhœa of Apis the blood is in strings, like small worms. The Apis diarrhœa seems to be due to an ulceration of Lieberkuhn's glands."

On the same evening in commenting on potencies, Dr. Fellger said that "our reason for believing in potencies is the same as in the case of the magnet. We cannot see the influence that passes from it to the bit of steel, to move it, yet we see the latter raised when the magnet is presented to it. Hence we believe in the magnetic state. The same idea holds with regard to potencies."

Reichenback proved the existence of the odic force by exhibiting the flames in a dark room, and even photographing them.

The positive pole gave a blue light, while the negative gave red. Water which has been magnetized with the positive pole is pleasantly sour; whilst water magnetized with the negative pole is bitter.

Dr. Lippe, at another meeting, on being asked for a remedy for toothache, worse at night, said, Mag-carb. has toothache coming on soon after getting to sleep. It wakes him up. The pain is dull and grinding, and compels him to get up and walk about the room.

Dr. Smith spoke of Phos., which has toothache coming on only at night. Acon. has a teasing, hacking cough, dry and harsh, worse at night. A case of severe otalgia, worse at night, puzzled me for a short time. I gave Puls., but it did no good. The patient was a reserved, unobservant man, and but little information could be extracted from him. Finally he said that he desired to cover his head from the air. Upon this indication Silicea was given and he got better immediately. The next night he was not forced to walk the floor.

Dr. Lippe said that a desire to cover the head from the air ran through Silicea as a characteristic. In answer to a question Dr. L. said that Kali bichromicum has the symptom vertigo on rising in the morning.

The characteristic of Petroselinum is frequent passing of urine. This is Hahnemann's key-note. Dr. Lippe used it in gonorrhœa when there is a great deal of burning with frequent passage of urine.

Dr. Smith gave Petros. and cured a child who, when urinating, would press hard as if pressing something solid out. It would rub the legs together until the skin came off. Dr. Lippe had a case of secondary syphilis with intense pain in the penis. He gave Nitric acid and the pain was relieved, but the patient complained of feeling as though splinters were sticking in the penis. Dr. L. then found that the chancre had been treated with crude Nitric acid.

Dr. Guernsey, on the same evening, speaking of diseases of the skin, said that Natrum muriaticum is useful where there is intense itching of the eruption. And that Agaricus musc. has a more noticeable action on the upper part of the body. He had used Bromine in some skin affections where there was coryza present with corrosion of the margins of the nose.

Dr. Lee described the case of a child whose condition was very obscure and where emaciation was prominent. He kicked constantly with his left foot until the stocking came off. Zinc. cured the case.

Dr. Smith described a case of pneumonia, a child, who had been given up to die by two allopaths. He found the child plunging about the bed as if it were swimming. He would dive in among the bedclothes after the manner of a porpoise. Sulphur was given and the child recovered.

One evening Dr. Lee read a paper on "Our Materia Medica." Dr. Lippe, commenting: How do we know how to cure any affection, fungus hematodes, for instance? Was fungus hematodes found in the proving of Phosphorus? No, Hahnemann gave us the symptom: small wounds bleed much. This is all the indication we have in some cases of that affection, and yet see what Phos. will do. As to the question of the dose, the greatest liberty of opinion should be held; that has nothing to do with our law. Cures can be made homœopathically with any dose, but the best are made with the high potencies. The smaller the dose of the perfectly similar remedy, and the longer it acts, the more lasting is the cure.

Dr. Clark observed that those who followed Hahnemann in practicing Homœopathy were the positive men, while those who were not familiar with his teachings, and yet assumed his name, were always in doubt, in a negative state, and would say the success in practice is luck. Dr. Lippe, in illustration of his remarks, related the case of an old lady æt. about eighty years, with paraplegia. On the indication: ineffectual effort at stool and dry tongue, he gave Nux moschata, and improvement immediately began.

Dr. Fellger told of an extraordinary case of an artist who had severe pain in pit of stomach and inability to talk; he can only whisper a few words, when he becomes exhausted. If he attempts to speak he is attacked by this pain. The sight of a picture which is not artistically correct, or a caricature, causes intense nervousness, profuse perspiration, and pain at pit of stomach. No remedy but magnetism had been of the least avail. Dr. Fellger thought it incurable. Dr. Lee suggested that his symptoms could be defined thus: trifles irritate him. Dr. Lippe did not think this would cover the case. He could not suggest a remedy, and agreed with Dr. Fellger that, it having continued so long, and no remedy relieving, it was incurable.

